# Atomic force microscopy visualization of injuries in Enterococcus faecalis surface caused by Er,Cr:YSGG and diode lasers

**DOI:** 10.4317/medoral.19991

**Published:** 2014-12-05

**Authors:** Lidia López-Jiménez, Josep Arnabat-Domínguez, Miguel Viñas, Teresa Vinuesa

**Affiliations:** 1 Laboratory of Molecular Microbiology and Antimicrobials. Dept. Pathology and Experimental Therapeutics; 2 Department of Dentistry. Medical and Dentistry schools. IDIBELL. University of Barcelona

## Abstract

Aim: To visualize by Atomic Force Microscopy the alterations induced on *Enterococcus. faecalis* surface after treatment with 2 types of laser: Erbium chromium:yttrium-scandium-gallium-garnet (Er,Cr:YSGG) laser and Diode laser. Material and Methods: Bacterial suspensions from overnight cultures of *E. faecalis* were irradiated during 30 seconds with the laser-lights at 1 W and 2 W of power, leaving one untreated sample as control. Surface alterations on treated *E. faecalis* were visualized by Atomic Force Microscopy (AFM) and its surface roughness determined. Results: AFM imaging showed that at high potency of laser both cell morphology and surface roughness resulted altered, and that several cell lysis signs were easily visualized. Surface roughness clearly increase after the treatment with Er,Cr:YSGG at 2W of power, while the other treatments gave similar values of surface roughness. The effect of lasers on bacterial surfaces visualized by AFM revealed drastic alterations. Conclusions: AFM is a good tool to evaluate surface injuries after laser treatment; and could constitute a measure of antimicrobial effect that can complete data obtained by determination of microbial viability.

** Key words:**Atomic force microscopy, Er,Cr:YSGG laser, diode laser, Enterococcus faecalis, surface roughness.

## Introduction

Biological diversity of oral microbiota is enormous in terms of number of species. A few of these bacteria can be isolated from root canal infections (1), post-treatment dental infections, and also from periodontal pockets (2). It has been reported that *Enterococcus faecalis* is the microorganism most commonly encountered in root canal infections after unsuccessful endodontic treatment (3,4). The main goals of many dental therapies (periodontal, endodontal, etc.) are to repair the affected region and to achieve the elimination of these microorganisms from root canals or crevicular region.

Several irrigant solutions, mechanical instrumentation and ultrasonic debridement are commonly used in the endodontic treatment (5-7). However, the morphology of root canal systems is extremely intricate and frequently these conventional treatments fail. Thus, it is difficult to guarantee the complete eradication of pathogenic bacteria in areas of difficult access such as fur cations and concavities (8). Similar difficulties can be claimed when looking at periodontal treatments. Recently, laser irradiations have been introduced into endodontic treatment as a suitable method to improve disinfection since its bactericidal effect has been well established (8,9).

The Erbium, Chromium:Yttrium-Scandium-Gallium-Garnet (Er,Cr:YSGG) laser and Diode laser, at wavelengths of 2,780 nm and 940 nm, respectively, are two different light sources widely used in dentistry. Dentinal tubules and enamel prisms can act as light conductors that keep the bactericidal effect of laser light and increase penetration depth. Previous studies have explored the anti microbial effect of both types of lasers. These reports were performed in different models of experimentally infected root canals (8,10,11). On the contrary, periodontal region seems to be inert to laser transmission.

Techniques such as Scanning Electron Microscopy (SEM), Con focal Laser Scanning Microscopy (CLSM) and Atomic Force Microscopy (AFM), are being used in dentistry research to characterize dentine, enamel and biomaterial surfaces, as well as to study the effect of laser treatments on tooth tissues and bacterial cells. SEM provides two-dimensional images of large areas of the surface, but is necessary previous dehydration and fixation that can affect the properties of the studied material. CLSM techniques give three-dimensional reconstructions of the subsurface of the samples with a similar resolution than SEM acquired images (12). AFM is a well-recognized powerful tool to visualize biological samples at the sub-molecular level achieving high-resolution images and the study of properties such as surface roughness; it also gives information concerning cell processes and interactions between microorganisms (13). Additionally, it does not need any special preparation of the sample that can produce alterations on the structure, moreover imaging in Non-Contact mode prevent any destruction of the sample. For this reason, AFM was used in this work to visualize damage on *E. faecalis* surface induced after treatment with Er,Cr:YSGG laser and Diode laser.

Growth conditions, nutrient availability, and environmental features inside the root canal are much more unfavourable for microbial growth than laboratory media, at least *ex vivo*. Thus, the widely used experimental strategy consisting in viable bacterial counts of survivors can in fact, underestimate the real antibacterial effect of laser light since injured individuals in optimal environmental conditions, such as those provided by laboratory rich media, can repair injuries and subsequently be able to form colonies whereas they would be unable to survive inside the canals. In this context, visualization of injuries caused in the bacterial surface could be a tool to estimate the level of damage and subsequently a useful approach to the clinical outcome of laser irradiation. Although sodium hypo chlorite (NaOCl), the most commonly used irrigant solution in endodontic treatment, seems to be a suitable method to exert bactericidal effect in root canals on the basis of bacterial counts, its toxicity for human cells should be taken into account. In this paper, using AFM techniques, we were able to obtain and compare imaging and roughness of microbial surfaces of a bacterium that causes most of the endodontic treatment failures.

## Material and Methods

- Bacterial strain and culture media

*E. faecalis*, American Type Culture Collection (ATCC) 29212 was used to study and compare the effectiveness of the two laser-light based treatments. Trypticase soy broth (TSB) was purchased from Scharlab (Barcelona, Spain). Distilled water was used to wash cells.

- Laser-light types

We used two different light sources. Er,Cr:YSGG laser system (Waterlase MD; Biolase Technology, Irvine, CA, USA) emits at a wavelength of 2,780 nm , in pulses of 140 and 750 ms, and a repetition rate of 10-50 Hz. Laser light is transferred from the source to the hand piece through optical fibre. The hand piece was an MVP (Biolase) and the tip was the radial firing tip (Waterlase MD Endolase RFT; diameter = 200 μm; length = 25 mm) designed for endodontic treatment (loss of power about 70%). Power was measured at the beginning using a watt meter (Coherent Inc., Santa Clara, USA). Tips were autoclaved before being used. 1 W and 2 W of power (display) were applied, at 20 Hz (20 pulses/s); thus, it was between 50 mJ and 100 mJ. Actual power was calculated on the basis of 30% efficacy (based on the diameter of the tip (200 μm), giving values of 0.3 W and 0.6 W, respectively (15 mJ and 30 mJ per pulse). The diode laser (InGaAsP; Ezlase, Biolase Technology, Irvine, CA, USA) emitting at a wavelength of 940 nm was also used. It can operate in pulsed or continuous mode and a repetition rate ranging from 0.06ms to 10 sec. It was provided of an endodontic tip (ezTip Endo, 14 mm / 200 μm). In this study we used it at 1 W or 2 W in pulsed mode with intervals of 1.0 ms and pulse length of 1.0 ms.

- Experimental procedures

Bacteria were grown in 10 ml of TSB medium at 37ºC overnight. The cultures were then centrifuged at 7000 rpm for 10 min in a Hermle centrifuge model Z 230 MA and then washed with distilled water. The tips of laser devices were submerged in the suspension and constant and helical movements were made during 30 sec. Irradiation was performed in micro tubes (PCR-02-A). The coaxial water spray and air were switched off. 1 W and 2 W of power of Er,Cr:YSGG laser and Diode laser were applied to 100 µl of the bacterial suspensions during 30 seconds. For cell immobilization and visualization under atomic force microscopy, 10 µl of treated and untreated bacterial suspensions were immediately transferred to mica Grade V-4 slides (SPI Supplies, USA) and air dried at room temperature in a dust-free environment.

- Atomic force microscopy visualizations

Samples were imaged by using an Atomic Force Microscope XE-70 (Park Systems, Korea). All images were collected in Non-contact mode by using pyramidal-shaped silicon cantilevers with a spring constant of ± 40 N/m and a resonance frequency of ± 300 kHz. The upper surface of these cantilevers (the opposite side of the tip) is coated with aluminum to enhance the laser beam reflectivity. The acquired data during the surface scanning were converted into images of topography, amplitude and phase; and analysed by using XEP and XEI software (Park Systems, Korea). The Non-contact mode provides topography, amplitude and phase images. On topography images it is possible to observe the shape, structure and differences of the sample surface. Furthermore, amplitude images highlight the outline and allow the visualization of fine surface details of the sample. Finally, the phase images show variations in elasticity and viscoelasticity of the sample (14). AFM images were simultaneously acquired with several scan sizes (100 µm2, 25 µm2 and 6.25 µm2) at a scan rate of 0.3-0.5 Hz.

- Surface roughness

AFM was also used to measure the surface roughness of treated and untreated cells. Roughness average (Ra) was calculated from the acquired topography images for every scan size and laser-light type. Ra represents the average distance from the roughness profile to the centre plane of the profile.

## Results

- Visualization of *E. faecalis* treated with Er,Cr:YSGG laser

**Figure 1:**
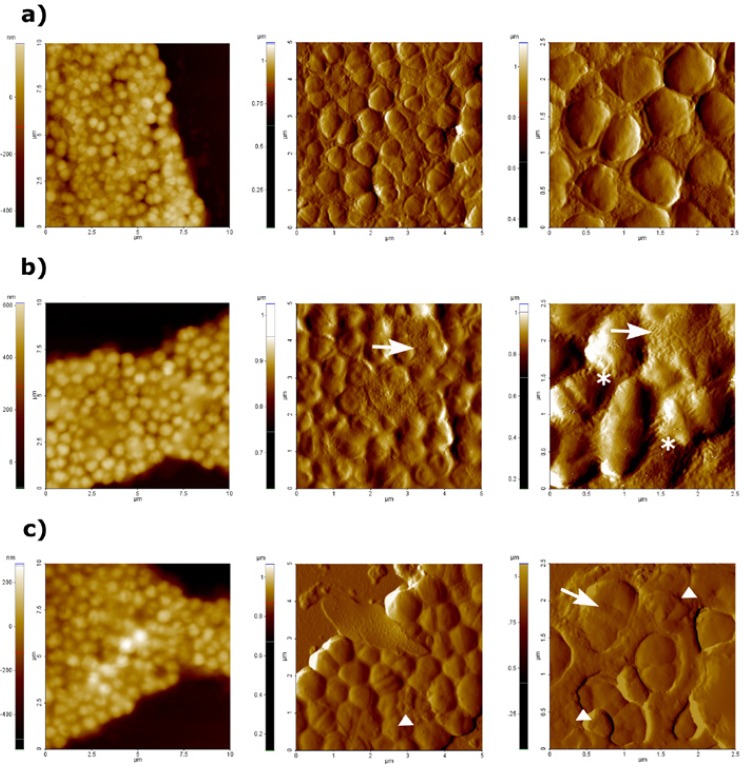
AFM images obtained at difference scan sizes (100 µm2, 25 µm2 and 6.25 µm2) of untreated E. faecalis (a), after treatment with Er,Cr:YSGG laser at 1W (b) and 2W of power (c). First column shows topography images, whereas amplitude images are shown in second and third column. Stretch marks are represented by asterisks, bacterial wall destruction or lose of typical cell morphology are represented by arrows, and leakage of intracellular content is represented by triangles.

**Figure 2:**
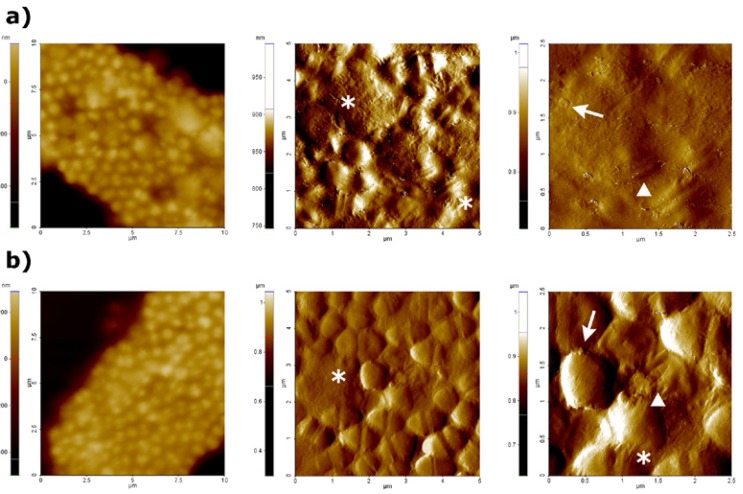
AFM images obtained at a difference scan sizes (100 µm2, 25 µm2 and 6.25 µm2) after treatment with Diode laser at 1W (a) and 2W (b) of power. First column shows topography images, whereas amplitude images are shown in second and third column. Stretch marks or lose of typical cell morphology are represented by asterisks, blebs are represented by arrows, and shape perturbations are represented by triangles.

AFM topography and amplitude images obtained after treatment of E. faecalis suspensions with Er,Cr:YSGG laser at both 1 W and 2 W, as well as AFM images of untreated bacteria are shown in figure 1. Topography images gave information about surface and structure differences whereas amplitude images allowed a better observation of surface characteristics and fine details (15). Comparing all AFM images, it was feasible to observe that after high potency of laser treatment, both cell morphology and surface resulted altered. Images of 100 µm2 size (Fig. 1 topography images) were acquired in order to obtain an overview of the sample, but do not provide relevant information since it was not possible to distinguish alterations in bacterial surfaces. On the contrary amplitude images of 25 µm2 and 6.25 µm2 sizes allowed the observation damages as cell morphology alterations and distortions on bacterial surfaces. Untreated *E. faecalis* cells appeared as typical coccoid shape (Fig. 1a). In contrast, changes in surface morphology and cell lysis signs were observed after treatment with Er,Cr:YSGG at 1 W (Fig. 1b) and 2 W of power (Fig. 1c), i.e. Stretch marks on the cell surface, bacterial wall destruction and lose of typical cell morphology. In addition to these signs, leakage of intracellular content was noticed on cells treated at 2 W of power (Fig. 1c), that indicates that Er,Cr:YSGG at this power could cause more damage on bacterial cells than at 1 W of power.

- Visualization of *E. faecalis* treated with Diode laser

Images provided by AFM microscopy of treated bacterial cells with Diode laser at 1 W and 2 W of power are shown in figure 2. Stretch marks on the cell surface, leakage of intracellular content and lose of typical cell morphology were observed as a result of both treatments. Moreover, in amplitude images of 6.25 µm2 scan size, some blebs and shape perturbations could be clearly appreciated along the bacterial cell surface; it seems feasible that this probably constitute another sign of bacterial lysis. AFM images showed that Diode laser could cause severe damages on E. faecalis cell surface, at both 1 W (Fig 2a) and 2 W (Fig. 2b) of power.

- Surface roughness

**Figure 3:**
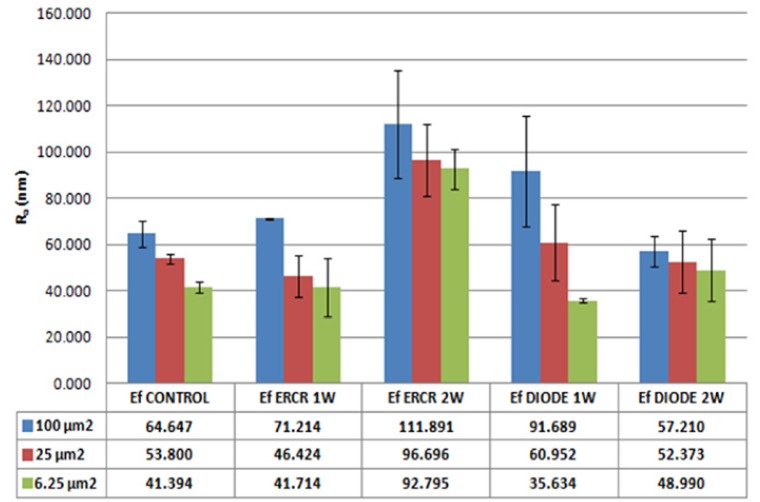
Graphical representations of surface roughness (Ra) in nanometers, according to the surface scan sizes, laser type and power used. Bars represented the standard error of the mean. Obtained values of mean surface roughness are shown in the table below.

In order to quantify the observed changes on bacterial cell surfaces treated, roughness measurements were performed by using the parameters provided by XEI software (Park Systems, South Korea) from topography images obtained previously at different scan sizes of 100 µm2, 25 µm2 and 6.25 µm2. Figure 3 represents mean surface roughness values (Ra) of untreated *E. faecalis*, and the same microorganism after treatment with Er,Cr:YSGG and with Diode laser, both of them at 1 W and 2 W of power. Analysing all obtained data, Er,Cr:YSGG laser at 2W of power produces the highest increase of surface roughness. By contrast, values of roughness obtained after the rest of laser treatments resulted to be similar as those obtained in untreated *E. faecalis*.

## Discussion

Microscopic techniques are being used in dentistry to characterize several types of surfaces and also to analyze quantitatively morphological changes on these surfaces after treatment. Dentine examination , visualization of root canals colonized by *E. faecalis*, evaluation of the effect of several laser radiations on root and enamel surfaces , and also the bactericidal effect of laser treatments have been studied by SEM (16-19). CLSM techniques have been used to analyse the subsurface of enamel and dentine after laser-light treatments (20,21). AFM has been recently introduced in dentistry as a sensitive and nondestructive tool for dentine surface characterization and quantification, to investigate mechanical properties of the dentine as well as to image dental tissue surfaces after laser-light treatments (16,22).

In this work, AFM was used to visualize the damage induced on *E. faecalis* surfaces after treatment with two different light sources, Er,Cr:YSGG laser and Diode laser. Both types of laser are being used in oral medicine and dermatology for surgical and anti microbial purposes. In 2010 we showed that Er,Cr:YSGG laser could be useful in dentistry to disinfect experimentally colonized root canals (11). Moreover, diode laser radiation gives similar results and, subsequently, is also useful for similar purposes (8). AFM allows visualizing of microbial surfaces both in air and in liquid. Imaging in liquid is more complicated from experimental point of view, at least in part, due to the poor adhesion of microbial cells to the substrate. This arise the noise during scanning and the resolution of obtained images is unsatisfactory (23). On the other hand, sample preparation for air imaging is easier and this mode of imaging reveals the overall morphology and topographic features of microbial cells surfaces that can be missed in liquid imaging (24). We have performed AFM measures in air as this mode of imaging is commonly used to evaluate morphological changes in microbial surfaces caused by several treatments (25,26). Moreover, imaging of untreated/control bacteria revealed neither morphological changes nor increases in nano roughness values.

When doing imaging by AFM of bacterial cell suspensions of E. faecalis after treatment with both lasers at different power (Fig. 1 and Fig. 2), it became clear that both lasers caused severe damage on bacterial surfaces as well as changes in bacterial morphology (size and shape). In addition to this, cell lysis signs could be observed in the AFM images acquired. Lee *et al*. (19), using SEM, observed morphological changes in *Streptococcus mutans* cells treated by a diode laser at several powers. Severe damages on bacterial cells were correlated with high laser powers; SEM acquired images revealed changes on lased bacteria such as loss of their wall bands, disintegration and fusion of cells with pores on the cell wall.

A relevant parameter to characterize the morphology of surfaces is the determination of the surface roughness (Ra), which represents the arithmetic average roughness (27). Surface roughness increased greatly after performing laser treatment. Moreover, a drastic effect on the shape was observed; i.e. bacterial cells appeared deformed and total lysis and leakage of cellular content could be seen (Fig. 1c, Fig. 2). This causes an increase in the surface roughness of the area that can be detected by AFM microscopy (Fig. 3).

When trying to correlate obtained AFM images, and surface roughness data with bacterial damaging, treatments with Er,Cr:YSGG laser at 2 W of power caused the most apparent morphological effect on bacteria. Specifically when comparing all AFM amplitude images of 25 µm2 and 6.25 µm2, we were able to observe remarkable changes on surface and morphology, as well as lysis signs in *E. faecalis* cells treated with Er,Cr:YSGG at 2 W. Surface roughness data were higher after this treatment as the other carried out in this study. Subsequently, one can assume that it could be the most effective treatment to eliminate and eradicate bacteria. The effects produced by treatment with Er,Cr:YSGG laser at 1 W, Diode laser at 1W and at 2 W of power, were almost identical in respect of surface and shape induced modifications.

These two lasers lights have the same photo thermal effect despite their differences in the interactions with the target tissue. Er,Cr:YSGG laser is strongly absorbed by water, whereas water absorbs diode laser at a lesser extent. Moreover it has a high affinity for hydroxiapatite, water is vaporized by a photo-thermal effect and the expansion of water vapour generates a photomechanical effect that could remove the smear layer on the dentinal surface, and also could disrupt intratubular bacteria (10). In contrast, Diode laser is poorly absorbed by tooth structures, the thermal effect of the radiation is transmitted in depth through the dentine with a low interaction with it, allowing a photo-disruptive effect on microorganisms present in the unreachable parts of the tubular network (28). The results obtained in the present study showed that Er,Cr:YSGG laser was more efficient in bacterial elimination than diode laser, despite several studies have reported higher bactericidal effect of the last one (29). A possible explanation for the better bactericidal effect of Er,Cr:YSGG laser found in this study could be related with the fact that all experiments were performed by using suspended bacteria, and the high water content of suspensions instead solid media may apparently enhance the Er,Cr:YSGG effectiveness.

When evaluating the anti microbial effect of light most authors have designed strategies on the basis of experimental colonization, followed by the recovery of microbes and enumeration of surviving bacterial populations. In all cases, cultivation of microorganisms to enumerate survivors is made in rich bacteriological media such as blood agar (30). The wide variety of complex nutrients from blood agar supports the growth of most bacteria facilitating the reparation of certain injuries which are not enough to induce cell death. However, growth conditions, nutrient availability, and environmental features inside the root canal are much more unfavorable for microbial growth. Thus, viable bacterial enumeration in artificial media can underestimate the real antibacterial effect of laser light, since to a certain extent bacterial cells with moderate damaging can repair lesions and multiply which probably will never happen into a root canal. In this study, we were able to visualize *E. faecalis* surface injuries by AFM just after treatment and perform a comparative study of microbial surface roughness. Our results are agree with previous studies, in which the effectiveness of Er,Cr:YSGG laser against *E. faecalis* in infected root canals was evaluated by cultivation to enumerate survivors. Wang *et al*. (19) reported that The reduction of *E. faecalis* inside root canals after treatment by Er,Cr:YSGG at 1 W and 2 W of power is 77% and 96%, respectively, moreover Er,Cr:YSGG at 2 W and 3 W of power is able to reduce the number of bacteria by 97.6% and 98.47%, respectively. According to the literature, the Er,Cr:YSGG laser at high power has shown an important bactericidal effect against *E. faecalis*.

## Conclusion

The AFM technique was useful to compare the efficiency of two types of lasers commonly used in dentistry. It provided visualizations of topographical and morphological changes on *E. faecalis* cells caused by laser irradiation, and also quantitative information of surface roughness. In this study, we can conclude that Er,Cr:YSGG at 2 W of power is the most effective in the extent of injuries and damages produced on bacterial cell surfaces. Since bacteria infecting root canals tends to form biofilm, further work to estimate the effect of Laser therapies on the integrity of bacterial biofilm is currently being developed. Further work is in progress to demonstrate similar damage evaluation in bacteria belonging to other taxa (Gram negative, Mycolata and others).
